# Analysis of Wild Type and Variant B Cystatin C Interactome in Retinal Pigment Epithelium Cells Reveals Variant B Interacting Mitochondrial Proteins

**DOI:** 10.3390/cells12050713

**Published:** 2023-02-23

**Authors:** Emil Carlsson, Umar Sharif, Wasu Supharattanasitthi, Luminita Paraoan

**Affiliations:** 1 Ocular Molecular Biology and Mechanisms of Disease Group, Institute of Life Course and Medical Sciences, University of Liverpool, Liverpool L69 3BX, UK; 2 Department of Physiology, Faculty of Pharmacy, Mahidol University, Nakhon Pathom 73170, Thailand; 3 Ocular Molecular Biology and Mechanisms of Disease Group, Department of Biology, Faculty of Arts and Sciences, Edge Hill University, Ormskirk L39 4QP, UK

**Keywords:** cystatin C, variant B, mitochondria, aging, mistrafficking, translocator protein, halo-tag, age-related macular degeneration, Alzheimer’s disease

## Abstract

Cystatin C, a secreted cysteine protease inhibitor, is abundantly expressed in retinal pigment epithelium (RPE) cells. A mutation in the protein’s leader sequence, corresponding to formation of an alternate variant B protein, has been linked with an increased risk for both age-related macular degeneration (AMD) and Alzheimer’s disease (AD). Variant B cystatin C displays intracellular mistrafficking with partial mitochondrial association. We hypothesized that variant B cystatin C interacts with mitochondrial proteins and impacts mitochondrial function. We sought to determine how the interactome of the disease-related variant B cystatin C differs from that of the wild-type (WT) form. For this purpose, we expressed cystatin C Halo-tag fusion constructs in RPE cells to pull down proteins interacting with either the WT or variant B form, followed by identification and quantification by mass spectrometry. We identified a total of 28 interacting proteins, of which 8 were exclusively pulled down by variant B cystatin C. These included 18 kDa translocator protein (TSPO) and cytochrome B5 type B, both of which are localized to the mitochondrial outer membrane. Variant B cystatin C expression also affected RPE mitochondrial function with increased membrane potential and susceptibility to damage-induced ROS production. The findings help us to understand how variant B cystatin C differs functionally from the WT form and provide leads to RPE processes adversely affected by the variant B genotype.

## 1. Introduction

Age-related macular degeneration (AMD) is the leading cause of blindness in the developed world [1], and is associated with the deterioration of the retinal pigment epithelium (RPE) monolayer of cells and loss of photoreceptors in the posterior of the eye [2,3]. In addition to age, environmental risk factors are commonly cited as drivers of disease onset and progression, alongside a significant genetic component of overall AMD risk [4]. Mutations across 34 locus regions are now attributed to approximately half of the overall genetic risk [5], with more rare mutations in other genes described as minor risk factors. One of these genes is *CST3*, encoding the protein cystatin C.

The 14 kDa cysteine protease inhibitor cystatin C is an abundantly expressed and directionally secreted protein from the RPE [6,7,8,9,10], where it is likely to regulate extracellular functions such as ECM remodeling through interactions with its molecular targets, the cathepsins in the eye. A single nucleotide polymorphism in the leader sequence of precursor cystatin C, encoding an A25T amino acid change resulting in a variant B protein, has been identified as a risk factor not only for AMD [11,12,13], but also Alzheimer’s disease (AD) [14,15,16]. The immediate functional effect of this mutation is not fully understood, but reduced secretion has been described from RPE cells as well as fibroblasts cells homozygous for variant B cystatin C [17,18,19,20]. Moreover, recently we applied an innovative CRISPR/Cas9 gene editing technique to create bi-allelic mutations in induced pluripotent stem cells (iPSCs) in order to express the AMD-linked variant B form of cystatin C under its endogenous promoter [21]. The resulting in vitro iPSC-differentiated RPE cells were shown to exhibit reduced cystatin C secretion, which led to an increase in RPE ability to degrade ECM material and migration capabilities. Furthermore, conditioned media from edited cells was able to stimulate the formation of significantly longer microvascular tubes compared to wild type conditioned media, which supports a role for angiogenesis relevant to AMD changes [22]. In addition, previous studies using live cell microscopy techniques have indicated that a fraction of the intracellular variant B cystatin C protein pool associates with mitochondria in cultured RPE cells [18,23]. This was highly unexpected, not predicted, and likely a consequence of its disturbed intracellular trafficking. The present study aimed to provide increased functional understanding of variant B cystatin C association with mitochondria.

An increasing number of studies have highlighted the maintenance of mitochondrial homeostasis as an essential process in promoting RPE cellular health with age. In general, cells undergo a decline in mitochondrial activity with aging [24]. However, dysfunctional mitochondria [25], mitochondrial DNA damage [26], up- and down-regulation of mitochondrial proteins [27,28,29,30], and mitochondrial disintegration [31] have also been observed in the RPE with AMD. However, the mechanisms underlying this behavior are poorly understood. Although mitochondrial modulation to promote proteostatic control in the RPE and retina in general has been suggested as a possible therapeutic avenue for managing AMD and other degenerative diseases [26], specific pathways are yet to be identified.

Given its unusual intracellular behavior, marked by an apparent mitochondrial association identified using visual analysis techniques, we hypothesized that variant B cystatin C may interact with one or more mitochondrial proteins that could have an impact on downstream mitochondrial function. Here we performed affinity purification mass spectrometry to elucidate and compare interactomes of wild type and risk-associated variant B cystatin C in RPE cells. Using Halo-tag technology, cystatin C fusion proteins were used as baits to capture interacting proteins on Halo-link resin, with eluates subjected to mass spectrometry analysis. We identified several proteins that exclusively bound to variant B cystatin C, including outer mitochondrial membrane (OMM) proteins, translocator protein (TSPO), and cytochrome B5 type B (CYB5B). In addition, analyses of mitochondrial functions revealed that the expression of variant B cystatin C in RPE cells resulted in alterations in susceptibility to mitochondrial ROS production and mitochondrial membrane potential (Δψm). This data provides for the first time information regarding the protein-protein interactions formed by the disease-linked variant B of cystatin C, and further demonstrates its impact on mitochondrial function.

## 2. Materials and Methods

### 2.1. Cell Culture

ARPE-19 cells (CRL-2302; ATCC) were cultured in Dulbecco’s Modified Eagle’s Medium/Nutrient Mixture F-12 Ham (DMEM:F12, Sigma, St. Louis, MO, USA) supplemented with 10% fetal bovine serum (FBS, Sigma, St. Louis, MO, USA) at 37 °C, 5% CO_2_. Upon reaching 80% confluency, cells were routinely passaged at a 1:3 ratio by dissociation with trypsin-EDTA solution (Sigma, St. Louis, MO, USA).

### 2.2. Plasmids

cDNA encoding WT or variant B cystatin C (encoding 1–146 aa full length protein) were subcloned into the pHTC HaloTag^®^ CMV-neo plasmid (Promega, Madison, WI, USA) between the EcoRI and XhoI restriction sites, using standard molecular biology techniques. Sequences were confirmed by Sanger sequencing (Source BioScience, Nottingham, UK). Preparation of plasmids encoding EGFP-tagged WT or variant B cystatin C has previously been described [8]. For mammalian cell transfections, all plasmids were propagated in DH5α and purified using an EndoFree Plasmid Kit (Qiagen, Hilden, Germany).

### 2.3. Expression of Halo-Tagged Cystatin C

Halo-tagged cystatin C was transfected in ARPE-19 cells by electroporation using the Neon system (Invitrogen, Carlsbad, CA, USA). For preparation of cell lysates for pull down assay, 1 × 10^7^ ARPE-19 cells were washed in DPBS and resuspended in 500 µL Neon resuspension buffer R. A total of 5 µg of plasmid was added to the mixture and electroporation was performed using 100 µL tips with the settings 1350 V, 20 ms, 2 pulses, after which the cells were immediately added to the culture medium. Cells were incubated in a T75 flask for 24 h, followed by washing once in ice-cold DPBS and harvesting by scraping.

Fluorescent visualization of Halo-tagged cystatin C was performed with HaloTag TMRDirect ligand (Promega, Promega, Madison, WI, USA) added to the culture medium of transfected cells following the manufacturer’s instructions.

### 2.4. Pull down Interaction Assay

ARPE-19 cells expressing Halo-tagged proteins were resuspended in 300 µL mammalian lysis buffer supplemented with protease inhibitor (Promega, Promega, Madison, WI, USA). Following 5 min of incubation on ice, the lysis mixture was homogenized by passing through a 25G needle 10 times. The sample was clarified by centrifugation at 14,000× *g* for 5 min, and the supernatant was diluted to 1 mL using TBS and added to 50 µL Halo-link resin (Promega, Promega, Madison, WI, USA) prewashed three times with wash buffer (TBS + 0.05% IGEPAL-630). Protein complexes were allowed to bind to the resin overnight at 4 °C using end-over-end mixing. The resin was washed three times in wash buffer and once in TBS, after which bound proteins were eluted in 50 µL 50 mM ammonium bicarbonate supplemented with 0.1% Rapigest SF (Waters, Milford, MA, USA) at 80 °C for 10 min. Eluted samples were stored at −80 °C until analyzed.

### 2.5. SDS-PAGE and Silver Stain

10 µL of eluted samples were mixed 1:1 in 2× SDS-PAGE sample buffer, boiled at 95 °C for 5 min, and resolved by SDS-PAGE on a 12% gel, followed by visualization by silver staining using a Pierce silver stain kit (Thermo Scientific, Waltham, MA, USA). Gels were imaged using a ChemiDoc XRS+ imaging system (Bio-Rad, Hercules, CA, USA).

### 2.6. nanoLC-ESI-MS/MS Analysis

Mass spectrometry experiments and analysis were performed by the Warwick proteomics research technology platform using similar methods as described previously [32,33,34]. Following trypsin digestion, peptides were separated using reversed phase chromatography on an Ultimate 3000 RSLCnano system (Dionex, Sunnyvale, CA, USA). Mobile phase buffers A and B were 0.1% formic acid in water and 0.1% formic acid in acetonitrile, respectively. Samples were loaded onto an Acclaim PepMap µ-precolumn cartridge 300 µm i.d. × 5 mm 5 μm 100 Å (Thermo Scientific, Waltham, MA, USA) equilibrated in 2% aqueous acetonitrile containing 0.1% trifluoroacetic acid for 8 min at 10 µL min^−1^, after which peptides were eluted onto an Acclaim PepMap RSLC 75 µm × 25 cm 2 µm 100 Å (Thermo Scientific, Waltham, MA, USA) at 300 nL min^−1^ by increasing the mobile phase B concentration from 4–35% over 72 min, then to 80% over 3 min, followed by a 15 min re-equilibration at 4%.

Eluting peptides were converted to gas-phase ions by means of electrospray ionization and analyzed on a Thermo Orbitrap Fusion (Q-OT-qIT, Thermo Scientific, Waltham, MA, USA). Survey scans of peptide precursors from 375 to 1575 *m*/*z* were performed at 120K resolution (at 200 *m*/*z*) with a 5 × 10^5^ ion count target. Tandem MS was performed by isolation at 1.2 Th using the quadrupole, HCD fragmentation with a normalized collision energy of 33, and rapid scan MS analysis in the ion trap. The MS^2^ ion count target was set to 1 × 10^4^ and the max injection time was 200 ms. Precursors with a charge state of 2–6 were selected and sampled for MS^2^. The dynamic exclusion duration was set to 40 s with a 10 ppm tolerance around the selected precursor and its isotopes. Monoisotopic precursor selection was turned on. The instrument was run in top speed mode with 2 s cycles.

### 2.7. Data Analysis and Protein Identification

The raw data was processed using MaxQuant engine (https://www.biochem.mpg.de/6304115/maxquant (accessed on 1 November 2022)) against Homo sapiens database (http://www.uniprot.org/ (accessed on 1 November 2022)) and the common contaminant database from MaxQuant. Peptides were generated from a tryptic digestion with up to two missed cleavages, carbamidomethylation of cysteines as fixed modifications, and the oxidation of methionines as variable modifications. Precursor mass tolerance was 10 ppm, and product ions were searched at 0.8 Da tolerances. 

Scaffold (TM, version 4.8.2, Proteome Software Inc., Portland, OR, USA) was used to validate MS/MS based peptide and protein identifications. Peptide identifications were accepted if they could be established at >95.0% probability by the Scaffold Local FDR algorithm. Protein identifications were accepted if they could be established at >95.0% probability and contained ≥2 identified peptides. Proteins that contained similar peptides and could not be differentiated based on MS/MS analysis alone were grouped to satisfy the principles of parsimony. Proteins sharing significant peptide evidence were grouped into clusters. Protein groups with significant total spectra intensity were determined according to the T-Test, using Benjamini-Hochberg multiple test correction and *p* < 0.1.

### 2.8. Subcellular Fractionation

Crude mitochondrial fractions from ARPE-19 cells expressing Halo-tagged WT or variant B cystatin C were isolated by differential centrifugation using a mitochondrial isolation kit (Thermo Scientific, Waltham, MA, USA; Cat. No. 89874), following the manufacturer’s instructions. The mitochondrial pellet was solubilized in 2 × SDS-PAGE sample buffer, and the mitochondrial and cytosolic fractions were analyzed by immunoblotting.

### 2.9. Immunoblotting

Following SDS-PAGE using 12% gels, proteins were transferred to a nitrocellulose membrane using standard molecular biology procedures. Primary antibodies used were against cystatin C (Abcam, Cambridge, UK; ab109508), GAPDH (Abcam, Cambridge, UK; ab8245), CYB5B (Thermo Scientific, Waltham, MA, USA; PA5-52482), TOM20 (Cell Signaling Technology, Danvers, MA, USA; 42406), TSPO (Abcam, Cambridge, UK; ab109497), IRAP (also known as leucyl-cystinyl aminopeptidase; Cell Signaling Technology, Danvers, MA, USA; 6918), and α-tubulin (Abcam, Cambridge, UK; ab4074). After blocking in 5% milk in TBS supplemented with 0.1% Tween-20 (TBST) and probing with primary antibody, membranes were washed three times with TBST and incubated with secondary antibody conjugated to HRP (Sigma, St. Louis, MO, USA; A0545 or A9044), and then washed for an additional three times with TBST and developed using either Radiance Plus chemiluminescent substrate (Azure Biosystems, Dublin, CA, USA) or SuperSignal™ West Pico PLUS Chemiluminescent Substrate (Thermo scientific, Waltham, MA, USA). Blots were imaged using a ChemiDoc XRS+ imaging system (Bio-Rad, Hercules, CA, USA). Band intensity was assessed via densitometry and normalized against indicated housekeeping protein, and was used as a semi-quantitative estimate of protein concentrations. 

### 2.10. Mitochondrial ROS Production Measurement

ARPE-19 cells seeded into 6-well plate (3 × 10^5^ per well) were transfected with 0.5 µg of either EGFP only, WT, or variant B cystatin C endotoxin free plasmid DNA using the Neon electroporation system (same parameters used as for Halo-tag plasmids). Mitochondrial ROS detection experiments were performed using a commercially available kit (Cayman Chemical, Ann Arbor MI, USA) following the manufacturer’s protocol. Briefly, 24 h post-transfection, ARPE-19 cells were detached from wells using trypsin, resuspended in media, and centrifuged at 400× *g* for 5 min. Cell pellets were then resuspended in 100 µL of cell-based assay buffer (Cayman Chemical, Ann Arbor MI, USA) and centrifuged again at 400× *g* for 2 min. Cell pellets were resuspended in 500 µL of 20 µM Mitochondrial ROS detection reagent (Cayman Chemical, Ann Arbor MI, USA) and incubated for 30 min at 37 °C. Cell suspensions were centrifuged again and pellets were washed with Hank’s Balanced Salt Solution (HBBS) and centrifuged at 400× *g* for 5 min (step repeated twice). The final cell pellet was resuspended in 500 µL of warm HBBS +/− 10 µM antimycin A reagent (Cayman Chemical, Ann Arbor MI, USA) and incubated for a further 1 h at 37 °C. Cell suspensions were processed for flow cytometry analysis for mitochondrial ROS detection using the BD Accuri C6^TM^ instrument (BD Biosciences, San Jose, CA, USA). A minimum of 4000 events were collected in the P1 gate for each condition. Samples were analyzed using the BD Accuri C6 program and were utilized to detect fluorescent intensity that correlated to ROS levels (FL2 channel) from transfected cells only (FL1 channel). Flow cytometry analysis, using the BD Accuri C6 Software^TM^ (BD Biosciences, San Jose, CA, USA), provided transfection efficiency measurements, but more importantly allowed for the selection of only transfected green cells (FL1 channel) from mixed non-transfected and transfected populations. Mitotracker Red FM staining was measured on the FL2 channel with fluorescent intensity numbers used for quantification.

### 2.11. Measurement of Mitochondrial Membrane Potential (Δψm) Using Mitotracker Red FM

1.5 × 10^6^ ARPE-19 cells were transfected with 2.5 µg of either EGFP only, WT, or variant B cystatin C endotoxin free plasmid DNA using the Neon electroporation system (as above). 24 h post-transfection, ARPE-19 cells were stained with 100 nM Mitotracker Red FM (Thermo scientific, Waltham, MA, USA), a dye whose uptake is dependent on mitochondrial membrane potential (Δψm) for 30 min after which flow cytometry analysis was performed. A minimum of 10,000 events were collected in the P1 gate for each condition. Samples were analyzed on the BD Accuri C6 Flow Cytometry System (BD Biosciences, San Jose, CA, USA) in the same manner as for mitochondrial ROS detection in order to select transfected green cells (FL1 channel) from the mixed population. Mitotracker Red FM staining was measured on the FL3 channel with fluorescent intensity numbers used for quantification.

## 3. Results

### 3.1. Expression of Halo-Tagged Proteins

**Figure 1:**
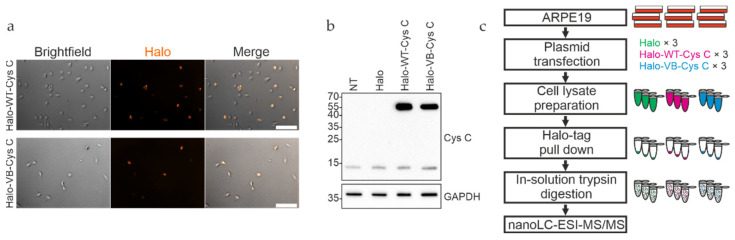
Expression and pull down analysis of Halo-tagged cystatin C in ARPE-19 cells. (**a**) WT and variant B (VB) cystatin C Halo-tag fusion constructs were expressed in ARPE-19 cells, followed by staining with Halo TMR Direct ligand and visualizing on a Zeiss Apotome fluorescence microscope. (**b**) Whole cell lysates of ARPE-19 cells expressing Halo-tagged WT and variant B cystatin C were analyzed by immunoblotting. Endogenously expressed cystatin C was present in all samples, alongside higher molecular weight Halo-tagged cystatin C present in the respective transfected cells. NT, non-transfected. (**c**) Schematic overview of workflow for pull down analysis of Halo-tagged fusion proteins.

To evaluate the different interaction partners of WT and variant B cystatin C, the two proteins were first expressed as Halo-tagged fusion proteins in ARPE-19 cells. The Halo-tag is a versatile fusion protein that allows irreversible attachment to a wide range of ligands while being biologically inert. In addition, successful expression can be confirmed directly by linking the tag to a fluorescent ligand. Expression constructs were generated by subcloning the full-length cystatin C protein coding sequence between the EcoRI and XhoI sites in the pHTC plasmid, resulting in a sequence-encoding cystatin C with a C-terminal Halo-tag attached. Positioning of the tag at the C-terminus allowed normal processing of precursor cystatin C, which involves cleavage of the N terminal signal sequence to form the mature protein. A high degree of expression of the respective proteins was achieved in transfected ARPE-19 cells (Figure 1a). Western blot analysis of cell lysates using an anti-cystatin C antibody showed bands consistent with the expected molecular weight of approximately 51 kDa for the Halo-cystatin C fusion proteins, in addition to the ~13 kDa endogenously expressed mature form of cystatin C (Figure 1b).

### 3.2. Interactome Analysis Reveals Specific Protein Interactions of Variant B Cystatin C with Mitochondrial Proteins

**Figure 2:**
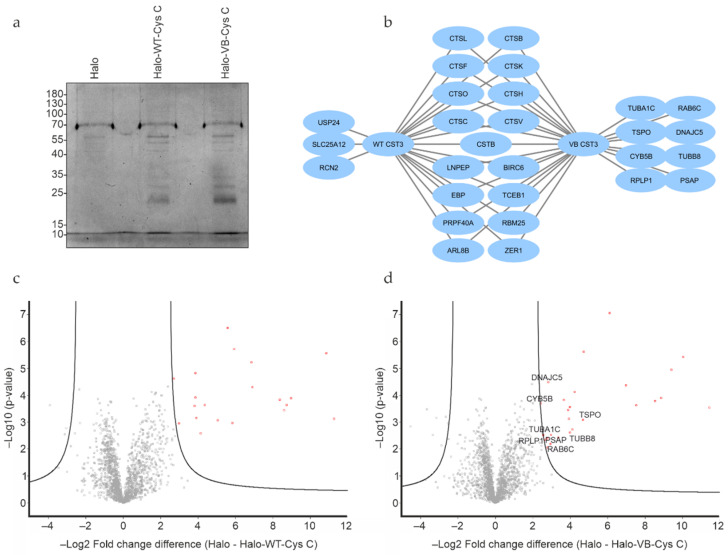
Protein interaction analysis of WT and variant B cystatin C by mass spectrometry. (**a**) Silver stain analysis of eluates from Halo-tag protein pull down experiments indicate multiple proteins eluted from pull down assays using both fusion constructs. (**b**) Visual representation of interacting proteins identified from Halo-tag pull down analysis using WT or variant B cystatin C fusion constructs. Volcano plots of mass spectrometry results of eluates from Halo-tag pull down analysis using WT (**c**) or variant B (**d**) cystatin C fusion constructs. Proteins exclusively pulled down by variant B cystatin C marked in plot. Cells transfected with plasmid encoding Halo-tag only were used as negative controls. All experiments were conducted in biological triplicates.

Proteins from ARPE-19 whole cell lysates interacting with either WT or variant B cystatin C fused to a C-terminal Halo-tag were isolated by immobilizing the bait proteins via their Halo-tag on Halo-link resin, followed by extensive washing to remove contaminants/non-interacting proteins and elution of interacting proteins in Rapigest buffer (Figure 1c). As Rapigest is fully compatible with downstream trypsin cleavage and mass spectrometry analysis, this method was preferred to the standard SDS elution buffer, which would require an intermediate buffer exchange step before analysis. Silver staining of eluates indicated that several proteins had been pulled down (Figure 2a), and mass spectrometry analysis was performed to identify proteins interacting with cystatin C, as well as discern any differences between WT and variant B interaction partners. A total of 29 individual proteins (including the bait cystatin C) could be detected in the samples. Summaries of the identified proteins are listed in Table 1 and Table 2 and shown diagrammatically in Figure 2b. Volcano plots were generated for eluted fractions from both bait proteins compared with the eluted fractions of a control sample from pull down analysis with cells transfected with empty pHTC plasmid (Figure 2c,d).

**Figure 3:**
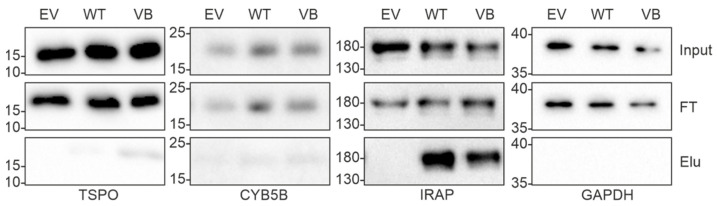
Validation of protein interaction analysis of WT and variant B cystatin C by immunoblotting. Immunoblot analysis of pull down samples from ARPE-19 cells transfected with empty vector (EV), wild-type cystatin C (WT), or variant B cystatin C (VB). Top panels show inputs, middle panels show flow through (FT), and bottom panels show eluates (Elu). GAPDH was used as a negative control. IRAP (encoded by *LNPEP*) was pulled down by both WT and variant B cystatin C. Blots shown are representative of three separate experiments.

As expected, the majority of interacting proteins, including most of the high abundance proteins, were similar across WT and variant B pull downs. These included cathepsins C, L, K, H, B, O, F, and V, which are known targets of cystatin C, providing confidence that the assay and analysis had worked as expected and that the Halo-fusion forms of cystatin C were active. However, a number of different interacting proteins were also identified. Of particular interest, among the eight proteins that were exclusively pulled down by variant B cystatin C, three (TSPO, CYB5B, and DnaJ homolog subfamily C member 5) are known to be abundantly distributed in mitochondria. Previous studies have indicated that a fraction of variant B cystatin C molecules mislocalize to the mitochondria [18], which may at least in part explain why reduced secretion of this protein occurs in RPE [18,22] and fibroblast cells [17]. Interaction between variant B cystatin C and TSPO was also confirmed via immunoblotting in separate, independent pull down experiments, while a much fainter band indicating a possible interaction with CYB5B was also seen (Figure 3). Notably, a minimal band for CYB5B could also be seen in the WT system, however, this was consistently of significantly lower intensity in relation to input, indicating a lower amount of protein pulled down by the WT protein and weaker interaction. Interestingly, clear interactions between IRAP and both WT and variant B cystatin C were also confirmed (Figure 3), consistent with a previous study showing cystatin C association with 3T3-L1 adipocyte GLUT4 storage vesicles [35], of which IRAP is a well-known component [36,37].

### 3.3. Expression of Variant B Cystatin C Leads to Alterations of RPE Mitochondrial Function

**Figure 4:**
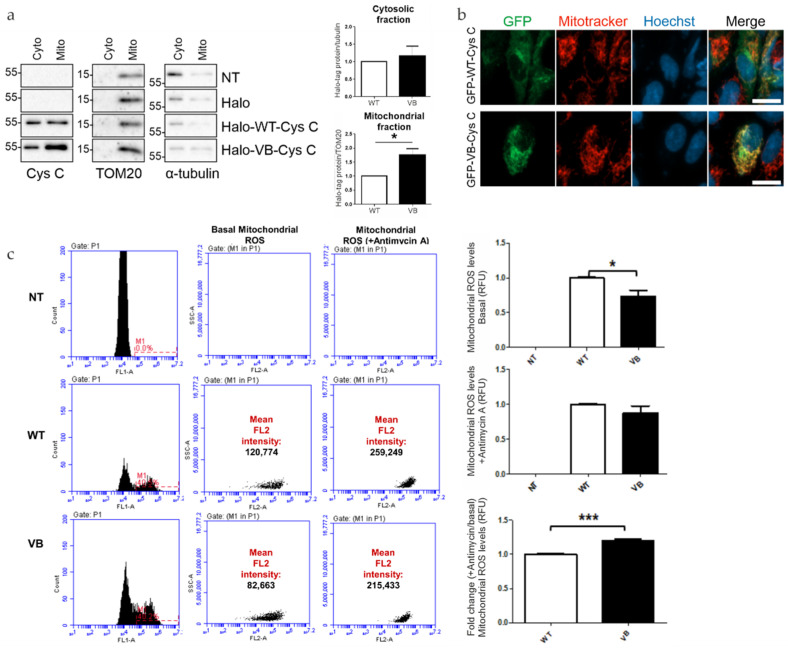
Analysis of mitochondrial association of variant B cystatin C and effects on mitochondrial reactive oxygen species (ROS) generation. (**a**) Immunoblot analysis of mitochondrial and cytoplasmic fractions of ARPE-19 cells expressing WT or variant B cystatin C Halo-tag fusion proteins. Blots were immunostained with antibodies against mitochondrial (TOM20) and cytoplasmic (α-tubulin) markers in addition to cystatin C. Blots shown are representative of three separate experiments. Graphs show normalized protein expression of Halo-tag fusion proteins in cytosolic and enriched mitochondrial fractions. (**b**) WT and variant B cystatin C EGFP fusion constructs were expressed in ARPE-19, followed by mitochondrial staining using Mitotracker Red FM dye and live cell analysis by fluorescence microscopy using a Zeiss Apotome microscope. Scale bars, 20 µm. (**c**) Mitochondrial ROS detection flow cytometry analysis in ARPE-19 cells transfected with WT and variant B cystatin EGFP fusion constructs (+/− antimycin A). After P1 gating was performed to exclude dead cells and debris (not shown), M1 (green channel) histograms were used to measure transfection efficiency (left of image). The M1 population was then selected to measure red intensity (FL2) as shown by plots (middle and right). Graphs show red fluorescence intensity (FL2 channel) in the transfected (green population; FL1 channel) cell population for basal mitochondrial ROS production (+/− antimycin A) and fold change between antimycin A treated cells/non-treated cells for each condition (arbitrary units ± S.E.M. minimum of *n* = 3; Student’s *t* test * *p* ≤ 0.05, *** *p* ≤ 0.001).

To test whether the Halo-tagged WT and variant B cystatin C differ in their subcellular localization when expressed in ARPE-19 cells, crude mitochondrial fractions were isolated from cytoplasm using differential centrifugation. Western blot analysis of the fractions confirmed the findings of previous studies showing association of a portion of variant B cystatin C pool and mitochondria by showing that the protein was enriched in the mitochondrial fraction in comparison to the WT protein (Figure 4a). Furthermore, expression of EGFP-tagged WT and variant B cystatin C in ARPE-19 was analyzed by fluorescence microscopy following staining with Mitotracker dye (Figure 4b), successfully replicating previously described results [18] with selected cells evidencing partial co-localization of variant B cystatin C and mitochondria, while WT cystatin C displayed a perinuclear enrichment typical of proteins processed through the classical secretory pathway.

After observing that variant B cystatin is significantly enriched in mitochondrial fractions compared to WT cystatin C, we next sought to see if the presence of variant B had an effect on mitochondrial function in relation to mitochondrial ROS production and Δψm. In relation to ROS production, a significant decrease in ROS production was observed in variant B-transfected RPE cells compared to WT cells (Figure 4c). Interestingly, upon the addition of antimycin A, an inhibitor of oxidative respiration that leads to a decrease in ATP production, similar levels of ROS were observed between WT and variant B cells, which resulted in a significant increase in fold change of mitochondrial ROS production (antimycin A/basal levels) for variant B-transfected RPE cells. These findings suggest that the presence of variant B at the mitochondria leads to increased susceptibility towards agents or stresses that compromise ATP production.

**Figure 5:**
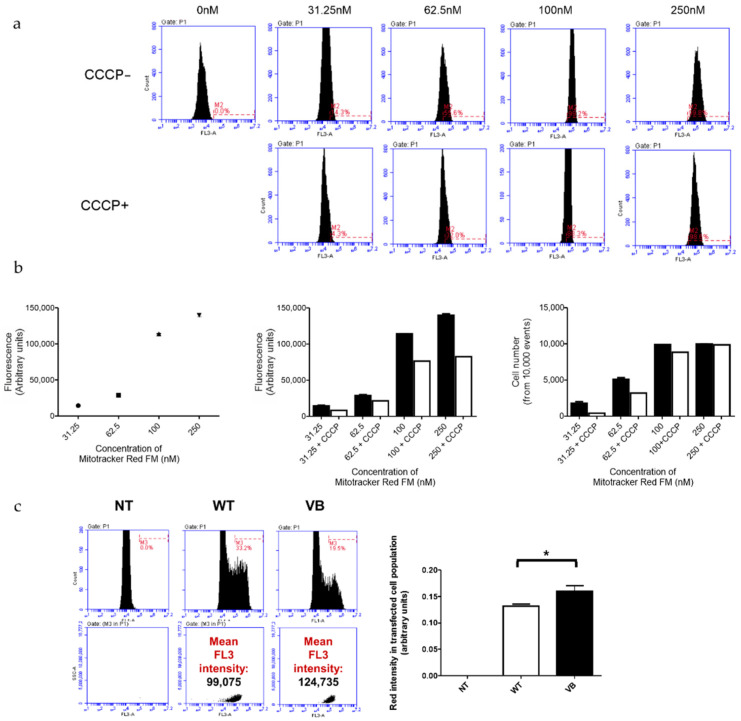
Analysis of mitochondrial membrane potential (Δψm) in ARPE-19 cells transfected with WT and variant B (VB) cystatin C EGFP fusion constructs. (**a**) Evaluation of optimal Mitotracker Red FM concentration required to stain the entire ARPE-19 cell population (+/− CCCP, an uncoupling agent that disrupts oxidative phosphorylation). Concentrations used for optimization ranged between 0–250 nM. Flow cytometry utilizing the FL3 channel was used for analysis. (**b**) Graphs show FL3 fluorescence intensity for cells stained with different concentrations of Mitotracker Red FM (left = optimization line graph; middle = +/− CCCP optimization bar graph; and right = number of cells stained with the concentration range of Mitotracker Red FM +/− CCCP). (**c**) Flow cytometry analysis of ARPE-19 cells transfected with EGFP WT or variant B cystatin and stained with Mitotracker Red FM. After P1 gating was performed to exclude dead cells and debris (not shown), M1 (green channel) histograms were used to measure transfection efficiency (left). The M1 population was then selected to measure red intensity (FL3), as shown by middle and right plots. Graph shows red fluorescence intensity (FL3 channel) in the transfected (green; FL1 channel) cell population (arbitrary units ± S.E.M. minimum of *n* = 3; Student’s *t* test * *p ≤* 0.05).

Next, in order to quantitatively evaluate the effects of variant B cystatin C on Δψm in RPE cells, the optimization of Mitotracker Red FM concentration was performed in order to use the dye in its sensitive range of concentration where differences can be detected. This showed that a concentration of 100 nM was sufficient to stain most cells whilst also allowing changes to be detected at a fluorescent and cell number level when CCCP, an uncoupling agent that disrupts oxidative phosphorylation, was added (Figure 5a,b). The presence of variant B cystatin C in RPE cells increased Δψm as indicated by increased fluorescence intensity readings in variant B-expressing RPE cells compared to WT transfected cells (Figure 5c). This data showed that mitochondria in RPE cells that contained variant B cystatin C existed in a functionally altered hyperpolarized state.

## 4. Discussion

In the present study, we have identified, for the first time to our knowledge, the interactome of WT cystatin C and AMD/AD-associated variant B cystatin C. The findings highlighted specific variant B cystatin C interacting mitochondrial proteins while also confirming the enrichment of variant B in mitochondrial fractions isolated from RPE cells expressing this form of the protein. Furthermore, we showed that the presence of variant B cystatin C in RPE cells leads to alterations in mitochondrial ROS production and Δψm. Thus, this study delineates possible routes on which variant B cystatin C interacts with mitochondrial processes and proteins, impacting mitochondrial function and contributing to mitochondrial dysfunction, which is a characteristic feature described for both AMD and AD.

Our mass spectrometry and pull down data showed that variant B cystatin C interacts with CYB5B and TSPO. This is of interest due to the fact that both CYB5B and TSPO are localized to the outer mitochondrial membrane, thus comprising plausible anchor points for mistrafficked cystatin C. TSPO has been previously highlighted for its potential role in neurodegeneration seen in AD [38]. While it is nearly absent in the healthy adult brain, it has been shown to accumulate at sites of senile plaques in an AD model [39], possibly as a form of defense mechanism. Additionally, administration of the small molecular TSPO ligand PK11195 has been reported to reduce soluble and deposited ß-amyloid [40]. Variant B cystatin C is inefficiently cleaved and/or processed, which has been proposed to result in an incompletely processed precursor protein [18] that has increased propensity to aggregate and form amyloid fibrils [41]. It is possible that aggregated variant B cystatin C attaches itself onto the mitochondria due to TSPO’s ability to associate with and modulate amyloid fibrils. Furthermore, TSPO is involved in other biological roles that may be compromised through its interaction with variant B cystatin C.

Recent studies have demonstrated other roles in a wide range of processes for TSPO, such as oxidative stress, calcium transport, mitochondrial function, apoptosis, inflammation, and perhaps most intriguing, autophagy, all of which have been linked to RPE dysfunction and AMD development [31,42,43,44,45,46,47,48,49]. Selective autophagy of the mitochondria, also known as mitophagy, is a key process in the elimination of damaged mitochondria [50]. Failure to remove these fragments leads to an unwanted accumulation inside the cell and subsequent cellular dysfunction, while uncontrolled activation of mitophagy pathways can elicit cell death. Impairment of mitophagy is theorized to play a role in AMD pathogenesis, and in this light, whether variant B cystatin C contributes to this effect via interaction with TSPO warrants further investigation.

The functional effects of variant B cystatin C enrichment at the mitochondria were investigated through measurement of mitochondrial ROS and Δψm levels. Expression of variant B cystatin C caused a decrease in basal mitochondrial ROS levels in RPE cells, but an overall increase in fold change of mitochondrial ROS levels upon the addition of antimycin A compared to cells expressing WT cystatin C. ROS are generally produced from the mitochondria and play an important role, either physiological or pathophysiological, directly dependent of their level, in a wide range of cellular processes such as cell survival/death and inflammation [51,52,53]. Excess ROS formation generally leads to a state of oxidative stress, causing the accumulation of ROS-associated damage in DNA, lipids, and proteins; such processes are believed to contribute to RPE dysfunction and development of AMD [54]. Increased levels of mitochondrial ROS promote production of pro-inflammatory cytokines in mouse embryonic fibroblasts and human immune cells harboring a missense mutation in type 1 TNF receptor (*TNFR1*), a change which causes an autoinflammatory disorder called tumor necrosis factor receptor-associated periodic syndrome (TRAPs) [55]. Furthermore, mitochondrial ROS have been linked to inflammasome activation [52]. It is possible that the initial decrease in mitochondrial ROS levels may be an adaptive protective response employed to protect variant B-expressing RPE cells against stresses encountered with age [56] that increase ROS levels and elicit inflammation.

The concept of protection is supported by findings in hypoxic cells, specifically that an adaptive response mediated by hypoxia-inducible factor 1 (HIF-1) reduces mitochondrial ROS production levels through multiple mechanisms, including increasing efficiency of electron transport chain (ETC) components [57]. It has been demonstrated that TSPO overexpression in *Jurkat* cells resulted in increased gene expression of mitochondrial ETC molecules and ATP production [58], thus evidencing TSPO involvement in mitochondrial energy metabolism. Although in need of further investigation, variant B cystatin C interaction with TSPO may compromise TSPO mitochondrial energy metabolism function and make these mitochondria less efficient at consuming oxygen. If this is the case, then it is plausible that undamaged mitochondria that do not contain variant B may increase ETC efficiency, which would result in reduced overall ROS levels [57].

Here we also identified an interaction between variant B cystatin C and OMM CYB5B, comprising an additional plausible site for ETC modulation. An overall increase in fold change mitochondrial ROS levels (upon the addition of antimycin A) in variant B-expressing cells compared to WT-expressing RPE cells suggests that the presence of variant B renders RPE cells more susceptible and responsive to ETC inhibitors such as antimycin A. In relation to RPE cells, one such stress known to accumulate with age in cells and in the Bruch’s membrane (BrM) is represented by advanced glycation end products (AGEs), a group of heterogeneous molecules that impact RPE function. AGEs have been shown to increase mitochondrial ROS [59], impair respiration, and target complex III of ETC, similar to antimycin A [60]. Therefore, with exposure to age-related stresses such as AGEs, it is possible that variant B cystatin C-expressing RPE cells would exhibit increased response in mitochondrial ROS production and subsequent processes such as inflammation. Indeed, RPE cells grown on AGE-containing matrixes have previously been shown to employ protective mechanisms that enable them to survive against the stress of AGE, but renders them more responsive to pro-inflammatory stimuli [61]. The implications of increased fold change mitochondrial ROS, exposure to age-related stresses such as AGE, and links to cell survival/inflammation definitely warrants further investigation in order to get a better understanding of cystatin C-mediated mechanisms contributing to RPE dysfunction and AMD progression.

In addition to altered ROS levels, an increase in ΔΨm was determined, indicating that variant B cystatin C-expressing RPE cells contain functionally altered hyperpolarized mitochondria. Hyperpolarized mitochondria have been observed in iPSC-derived neurons isolated from patients carrying a mutation in the Tau gene that causes the neurodegenerative disease frontotemporal dementia with parkinsonism linked to chromosome 17 (FTDP-17) [62]. This hyperpolarized mitochondrial state of neurons was shown to lead to overproduction of mitochondrial ROS, which in turn caused oxidative stress and cell death, events also linked with RPE dysfunction and AMD progression.

Generally, a positive correlation exists between ΔΨm and mitochondrial ROS production [63]. However, in the present study, an initial decrease in mitochondrial ROS levels was observed in variant B-expressing RPE cells, along with an increase in ΔΨm. Similar to the present study, in vitro human fibroblasts treated with nicotinamide (NAM), a form of vitamin B_3_ with antioxidant effects, displayed lowered mitochondrial ROS and increased ΔΨm as well as, intriguingly, extended cell life span [64]. This extension of lifespan, which was suggested to be due to decreased mitochondrial activity, is notable, as it supports the idea of variant B cystatin C-expressing RPE cells reducing ROS and increasing ΔΨm as a potential protective mechanism. Furthermore, NAM-treated cells displayed lower mitochondrial content through increased mitophagy [65]. It was initially hypothesized that mitophagic removal of high ROS producing, low depolarized mitochondria is the cause of overall presence of mitochondria displaying decreased ROS levels and increased ΔΨm in NAM-treated cells. However, a more recent study demonstrated that the NAM-induced reduction in mitochondrial ROS and increase in ΔΨm was due to decreasing electron flow through complex I of ETC, which led to decreased oxygen consumption and mitochondrial ATP production, as well as blockage of the mitochondrial permeability transition pore (mPTP), respectively [66].

Opening and closing of mPTP formed on the inner mitochondrial membrane is involved in regulating calcium levels that are driven by ΔΨm [67]. Opening this channel is linked with a large efflux of calcium and is often linked to cell death in diseases such as AD [68]. Taken together, it could be that decreased ROS levels and increase in ΔΨm caused by the presence of variant B cystatin C may be indicative of RPE cells protecting themselves. This protection may involve increased mitophagy, decreased mitochondrial activity, which is linked to extended lifespan, and closure of the mPTP channel to reduce cell death. This, in the short term, may be beneficial to help compensate for variant B-associated mitochondrial dysfunction, but in the long term, constant decreased mitochondrial activity would be detrimental to the functional output of these highly metabolic post-mitotic cells. The effects of variant B cystatin C on the ETC, mitochondrial activity, and processes such as mitophagy in in vitro models of aging are currently being addressed in our laboratory and will provide a better understanding of how this mutant contributes to RPE dysfunction and AMD development.

Limitations of this study include the use of the ARPE19 cell line as opposed to using primary or iPSC-derived RPE tissue, as well as allowing cells to go into suspension for electroporation instead of allowing them to form confluent and polarized monolayers. Although such alternative systems may have provided a higher level of physiological relevance, the choice of ARPE19 cells was founded on the critical need to achieve high levels of transient expression of fusion protein in a well-characterized model system. It is therefore important that future studies focus on validating results in primary tissue to understand the mechanistic relationship between variant B cystatin C and AMD development. Furthermore, although our experiments indicate a functional effect of variant B cystatin C on mitochondria, the exact mechanism and conditions involved need to be assessed in future studies to fully understand their impact on mitochondria and link with age-related diseases.

In conclusion, we identified for the first time mitochondrial proteins that interact with variant B cystatin C. In addition, our data shows that the presence of variant B cystatin C impacts mitochondrial function, as observed through decreased mitochondrial ROS production and increased ΔΨm. Notably, other models that show similar results suggest these mechanisms are indicative of protection mechanisms for the cells. However, fold change mitochondrial ROS data (upon the addition of antimycin A) suggests that variant B cystatin C-expressing cells are more susceptible to detrimental ETC modulating stresses such as AGE, which accumulate with age.

## Figures and Tables

**Table 1:** Proteins pulled down by WT cystatin C.

Identified Protein	Gene Name	Uniprot ID	*p* Value (-LOG10)	Difference (LOG2)	Unique Peptides	Unique Sequence Coverage (%)	Molecular Weight (kDa)
Cathepsin C	*CTSC*	P53634	3.128994029	11.26671028	15	39.1	51.853
Cathepsin L	*CTSL*	P07711	3.885627129	8.967143377	6	25.2	37.564
Cathepsin K	*CTSK*	P43235	3.642980692	8.719130198	10	50.5	36.966
Cathepsin H	*CTSH*	P09668	3.443870854	8.583513896	9	31.6	36.269
Cystatin B	*CSTB*	P04080	3.834472823	8.368869781	5	77.6	11.139
Ubiquitin carboxyl-terminal hydrolase 24	*USP24*	Q9UPU5	4.302084411	6.91021347	2	0.8	294.36
Cathepsin B	*CTSB*	P07858	5.215647829	6.847566605	18	50.7	37.821
Cathepsin O	*CTSO*	P43234	5.722981088	5.925343196	6	18.4	35.957
Leucyl-cystinyl aminopeptidase	*LNPEP*	Q9UIQ6	2.964108501	5.812737147	26	25.6	117.35
Protein zer-1 homolog	*ZER1*	Q7Z7L7	6.50224812	5.582649867	19	26.1	88.169
Baculoviral IAP repeat-containing protein 6	*BIRC6*	Q9NR09	3.072196881	5.047833761	21	6.5	530.25
Cathepsin F	*CTSF*	Q9UBX1	3.643260716	4.351509094	5	15.3	53.365
Transcription elongation factor B polypeptide 1	*TCEB1*	Q15369	2.590215898	4.142413457	5	70.8	9.9601
Emopamil-Binding Protein	*EBP*	Q15125	3.145526514	3.908337275	3	16.5	26.352
Pre-mRNA-processing factor 40 homolog A	*PRPF40A*	O75400	4.823116829	3.851610184	8	11.3	108.8
ADP-ribosylation factor-like protein 8B	*ARL8B*	Q9NVJ2	3.917623907	3.847599665	3	13.4	21.539
Cathepsin V	*CTSV*	O60911	3.596574475	3.820123037	3	14.1	37.329
Reticulocalbin-2	*RCN2*	Q14257	2.957438588	2.969583511	8	35.6	36.876
RNA-binding protein 25	*RBM25*	P49756	2.47628457	2.762470245	9	15.1	100.18
Solute Carrier Family 25 Member 12	*SLC25A12*	O75746	4.617153607	2.696769714	10	18	74.761

**Table 2:** Proteins pulled down by variant B cystatin C.

Identified Protein	Gene Name	Uniprot ID	*p* Value (-LOG10)	Difference (LOG2)	Unique Peptides	Unique Sequence Coverage (%)	Molecular Weight (kDa)
Cathepsin C	*CTSC*	P53634	3.542433544	11.42620722	15	39.1	51.853
Cathepsin L	*CTSL*	P07711	4.944991294	9.40882047	6	25.2	37.564
Cystatin B	*CSTB*	P04080	3.90733015	8.848660151	5	77.6	11.139
Cathepsin K	*CTSK*	P43235	3.778308165	8.52559344	10	50.5	36.966
Cathepsin H	*CTSH*	P09668	3.620166418	7.520583471	9	31.6	36.269
Cathepsin B	*CTSB*	P07858	4.369058919	6.979846319	18	50.7	37.821
Cathepsin O	*CTSO*	P43234	7.050883497	6.113189697	6	18.4	35.957
Protein zer-1 homolog	*ZER1*	Q7Z7L7	5.61326774	4.726226807	19	26.1	88.169
Translocator protein	*TSPO*	P30536	3.089099339	4.6859773	3	36.4	11.896
ADP-ribosylation factor-like protein 8B	*ARL8B*	Q9NVJ2	4.124792469	4.229539871	3	13.4	21.539
Baculoviral IAP repeat-containing protein 6	*BIRC6*	Q9NR09	2.727875293	4.118724187	21	6.5	530.25
Emopamil-Binding Protein	*EBP*	Q15125	3.565297847	3.988709768	3	16.5	26.352
Tubulin beta-8 chain	*TUBB8*	Q3ZCM7	2.606538895	3.97833252	2	7.4	49.775
Cathepsin F	*CTSF*	Q9UBX1	3.123290332	3.924125671	5	15.3	53.365
Cathepsin V	*CTSV*	O60911	3.457522376	3.883289973	3	14.1	37.329
Pre-mRNA-processing factor 40 homolog A	*PRPF40A*	O75400	3.826395659	3.662123362	8	11.3	108.8
Leucyl-cystinyl aminopeptidase	*LNPEP*	Q9UIQ6	2.430111396	3.348843892	26	25.6	117.35
Prosaposin	*PSAP*	P07602	2.525074435	2.975476583	14	28.3	58.44
Ras-related protein Rab-6C	*RAB6C*	Q9H0N0	2.182205955	2.939130147	2	12.2	28.242
DnaJ homolog subfamily C member 5	*DNAJC5*	Q9H3Z4	4.486646762	2.830143611	2	24.2	22.149
RNA-binding protein 25	*RBM25*	P49756	2.382926143	2.787614187	9	15.1	100.18
Transcription elongation factor B polypeptide 1	*TCEB1*	Q15369	2.059233321	2.78107961	5	70.8	9.9601
60S acidic ribosomal protein P1	*RPLP1*	P05386	2.349200506	2.666696548	2	51.8	11.514
Tubulin alpha-1C chain	*TUBA1C*	Q9BQE3	2.518650635	2.564816793	4	13.1	57.73
Cytochrome b5 type B	*CYB5B*	O43169	3.694849566	2.431503296	2	18	16.694

## Data Availability

The data presented in this study are available on request from the corresponding author.
